# Characteristics of PM_2.5_ and Its Correlation with Feed, Manure and NH3 in a Pig-Fattening House

**DOI:** 10.3390/toxics10030145

**Published:** 2022-03-19

**Authors:** Shihua Pu, Siyi Peng, Jiaming Zhu, Zuohua Liu, Dingbiao Long, TengTeeh Lim

**Affiliations:** 1 Chongqing Academy of Animal Sciences, Changlong Avenue, Rongchang District, Chongqing 402460, China; push@cqaa.cn (S.P.); engyi199512@email.swu.edu.cn (S.P.); zhujm@cqaa.cn (J.Z.); liuzh@cqaa.cn (Z.L.); 2 Scientific Observation and Experiment Station of Livestock Equipment Engineering in Southwest, Ministry of Agriculture and Rural Affairs, Chongqing 402460, China; 3 Innovation and Entrepreneurship Team for Livestock Environment Control and Equipment R&D, Chongqing 402460, China; 4 College of Animal Science and Technology, Southwest University, Chongqing 402460, China; 5 Division of Food Systems and Bioengineering, University of Missouri, Columbia, MO 65211, USA

**Keywords:** particulate matter, air pollutants, potential sources, primary particles, secondary particles

## Abstract

Fine particulate matter (PM), including PM_2.5_ in pig houses, has received increasing attention due to the potential health risks associated with PM. At present, most studies have analyzed PM_2.5_ in Chinese pig houses utilizing natural ventilation. These results, however, are strongly affected by the internal structure and regional environment, thus limiting their applicability to non-mechanically ventilated pig houses. This experiment was carried out in an environmentally controlled pig house. The animal feeding operation and manure management in the house were typical for Southwest China. To reduce the influence of various environmental factors on PM_2.5_, the temperature and humidity in the house were maintained in a relatively stable state by using an environmental control system. The concentration of PM_2.5_ in the pig house was monitored, while the biological contents and chemical composition of PM_2.5_ were analyzed, and feed, manure, and dust particles were scanned using an electron microscope. Moreover, bacterial and fungal contents and some water-soluble ions in PM_2.5_ were identified. The results showed that the concentration of PM_2.5_ in the pig house was strongly affected by pig activity, and a phenomenon of forming secondary particles in the pig house was found, although the transformation intensity was low. The concentration of PM_2.5_ had negative correlations of 0.27 and 0.18 with ammonia and hydrogen sulfide, respectively. Interestingly, a stronger correlation was observed between ammonia and hydrogen sulfide and ammonia and carbon dioxide concentrations (the concentration of ammonia had stronger positive correlations with hydrogen sulfide and carbon dioxide concentrations at +0.44 and +0.59, respectively). The main potential sources of PM_2.5_ production were feed and manure. We speculate that manure could contribute to the broken, rough, and angular particles that formed the pig house PM_2.5_ that easily adhered to other components.

## 1. Introduction

With the rapid expansion of intensive breeding in China, the number of enclosed and high-density pig houses has significantly increased, resulting in the production of a large amount of particulate matter (PM) in the environment. The particle size often determines the transmission distance between the particle and the microorganism, the suspension time, and the location of the deposition in the respiratory tract. Compared with large particles, PM with an aerodynamic diameter of less than 2.5 μm (PM_2.5_) can directly enter the alveoli and blood circulation via breathing due to their small size and large specific surface area, which also render them with the ability to easily adsorb harmful substances. PM_2.5_ particles are considered the most harmful particles to the body [1].

There are large differences in the morphology of PM_2.5_ in animal breeding houses. Particle surfaces carry a variety of heavy metals, water-soluble ions, microorganisms, and other harmful substances [2,3]. These substances can further endanger the health of the animals, since PM_2.5_ is a carrier of these types of substances. If the body is exposed to high concentrations of PM_2.5_ for a long time, chronic cough, chronic bronchitis, asthma, and other respiratory diseases may occur [4]. In addition, due to a series of innate immune reactions, the body’s resistance to other diseases and self-healing power are significantly decreased [5,6,7]. In addition, removal of PM_2.5_ from pig houses with ventilation can pollute the surrounding environment of pig farms and increase the chances of contracting respiratory diseases and health burden of nearby residents, potentially producing asthma and allergic rhinitis in children and adults [8].

At present understanding of the morphology, composition, and production of PM_2.5_ in Chinese pig houses is relatively limited, and current research is mainly conducted by monitoring or collecting samples in ventilated environments [9,10]. This system can affect the concentration and composition of PM_2.5_ in the houses due to the changes in natural and man-made factors outside the house. The relevant research focuses on the correlation between compound environmental factors and PM_2.5_, but these approaches may lead to unclear results due to the large changes in many factors. Few reports concerning the regulation of environmental parameters within relatively stable values can be found. Temperature is one of the most important environmental indicators in pig houses. Li et al. [11] found that the temperature of an animal house influences the changes in particle concentration. Fan et al. [12] considered that temperature and relative humidity play a central role in the distribution of fungi and bacteria (including pathogens) in PM_2.5_. The temperature changes can affect photochemical reactions in the house and thereby affect the content of water-soluble ions in PM_2.5_. It is thus hypothesized that after adjusting the temperature of pig houses to a relatively stable state, the characteristics of PM_2.5_ components and potential sources of PM_2.5_ in pig houses in China can be better studied and understood.

This experiment was carried out in an animal house with a controlled environment. Using negative pressure exhaust to ensure minimum proper ventilation, the temperature in the house was adjusted to a relatively stable level. The effects of the external environment and day–night temperature variation on PM_2.5_ were eliminated to a large extent. The objectives were to monitor indices of air pollution (ammonia [NH_3_], hydrogen sulfide [H_2_S], carbon dioxide [CO_2_]), and temperature, humidity, and PM_2.5_ concentration in a Southwest China pig house to examine the changes in the characteristics of PM_2.5_ and its correlation with the above environmental variables at a stable temperature. Furthermore, the study aimed to determine composition of PM_2.5_, feed, manure, and dust, and identify sources of PM_2.5_ production. The results are expected to provide a reference basis for better understanding of the changes in PM_2.5_ concentrations in pig houses and guide the precise regulation and control of pig house environments.

## 2. Materials and Methods

### 2.1. Description of The Pig House

The experiment was carried out in an environmentally controlled pig house (area 6 m × 9 m) at the Southwest Breeding Project Experimental Station of the Ministry of Agriculture. The experimental room was established in 2017 and located in Rongchang District, Chongqing City, China (East longitude 105°32′52.8″, North latitude 29°23′6″). The local annual average precipitation is 1099 mm, and the annual average temperature is 17.8 °C. The area has a typical subtropical monsoon humid climate. Six fenced pens (3.08 m long, 1.94 m wide, and 1.00 m high) were installed inside the pig house. Partially slatted flooring (1.97 m × 1.50 m) was installed within the fence, and liquid manure could enter a waste collection channel below.

**Figure 1:**
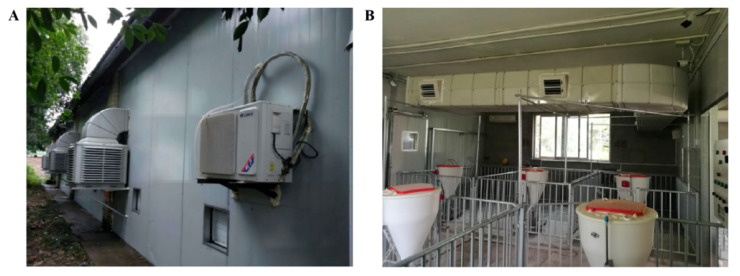
Environmentally controlled experimental pig house with thermal insulation and air conditioning units. (**A**) Ambient wall and external equipment. (**B**) In-house temperature control equipment and breeding environment.

The outer wall of the experimental room was thermally insulated with 75-mm interlayer colored steel rock wool to increase the thermal insulation performance of the pig research house and to reduce energy loss (Figure 1A). One air cooler, four exhaust fans, two inverter-mounted heating and cooling air conditioners, and two spoiler fans were installed in the cubicle. The air cooler was used for auxiliary ventilation and cooling (Figure 1B). The air from the air cooler was fed into the pig research house. The size of the air supply duct was 650 mm × 450 mm. The air supply duct was equipped with two air outlets (245 mm × 255 mm) on each side along the aisle. During the experiment, the exhaust fan was turned on for negative pressure ventilation, and the temperature of the room was regulated through a heating and cooling air conditioner. The spoiler fan assisted in accelerating the air flow in the test pig house to achieve the wind speed suitable for pigs in a controlled environment. The heaters and temperature switches were used to compensate for changes in the temperature of the experimental pig house at night, thereby reducing the temperature difference between day and night in the pig house.

### 2.2. Pig Management

Before the experiment, the house was cleaned and disinfected. The air conditioner was used to stabilize the temperature at about 26 °C. Eighteen Duroc × Landrace × Yorkshire (DLY) pigs (three in each pen, weight 70 ± 5 kg) that were routinely immunized and dewormed were introduced. After one week of acclimation, the experiment was conducted from 16 to 23 March 2021. During the experiment, dry pellet feed (No. 930 feed for pigs, Chongqing Huiguang Feed Co., Ltd., Chongqing, China) was supplied every day at 8:30 and 15:00 with free access to drinking water. The equipment in the house and the health of the pigs were regularly checked. Solid manure was removed manually at 8:45 each day, and the floor panel and liquid manure channels were washed after the experiment ended.

### 2.3. Measurement of Microclimate Variables

A hygrothermograph (HOBO U23-001, Onset, Bourne, MA, USA) was placed at a height of 1.5 m at the beginning and end of the walkway. The device recorded the temperature and humidity information every 5 min during the experiment. A dual gas path air sampler based on the principle of electrochemistry (KDY-C, Yancheng Keyuan Electronic Instrument Co., Ltd., Jiangsu, China) was placed at a height of 1.5 m in the middle of the house to monitor NH_3_, H_2_S, and CO_2_ concentrations in real-time, recording concentrations once every 10 s in the morning (7:00–10:00), afternoon (14:00–17:00) and night (19:00–21:00) each day during the experiment. The PM_2.5_ concentration was continuously monitored at a height of 1.5 m in the middle of the house using an oscillatory balance (Met One, Grants Pass, OR, USA) and recorded every 5 min.

### 2.4. Sample Collection and Component Analysis

An ambient PM collector (2030 type medium flow intelligent TSP sampler, Qingdao Laoshan Institute of Applied Technology) was placed at the beginning and end of the aisle to monitor concentration of PM_2.5._ The flow rate was set to 100 L∙min^−1^. The sampler was about 1.5 m from the ground, and the filter membrane was replaced every 24 h. The filter membrane of the collected sample was immediately wrapped with sterile aluminum foil, placed in an ice box, and returned to the laboratory for storage at −80 °C. A quartz fiber membrane (MK360, 90 mm) wrapped in aluminum foil was placed in a muffle furnace and heated to 500 °C for 5 h to remove residual organic matter and other impurities on the membrane. After treatment, the membrane was dried and stored for later use. During the experiment, about 1 kg of fresh manure, feed, and aisle dust were collected from the house, sent to the laboratory for mixing, and dried at 60 °C. After being ground into a fine powder in a blender, the samples were filtered with a 0.25-mm mesh polyvinylchloride (PVC) sieve to determine the contents of heavy metals (copper, zinc, chromium, cadmium, and lead [Cu, Zn, Cr, Cd, and Pb, respectively]).

The quartz filter membrane was cut to a size of 1 cm^2^ and placed in a sterilized 250 mL beaker. Then, 100 mL of high-purity water (18.2 MΩ) was added. The beaker was sealed with a sterile membrane and placed in an ultrasonic cleaner for 30 min, and this was repeated three times. Next, a 0.2-μm filter membrane was used for filtration (50 mm, Tianjin City Jinteng Experimental Equipment Co., Ltd., Tianjin, China). The filtrate was analyzed using a Hach instrument (DR6000, Hach, Loveland, CO, USA) and corresponding reagents for sulfate, nitrate, phosphate, and ammonium ion concentrations, and the filter membrane was used for subsequent microbial component analysis.

About half of the filter membrane was cut to a small piece of 1 cm^2^ and placed in a microwave digestion tube. A mixture of 6 mL concentrated nitric acid (HNO_3_), 2 mL hydrogen peroxide (H_2_O_2_), and 0.1 mL hydrogen fluoride (HF) was added to immerse the filter membrane, and the tube was capped and placed on a microwave turntable (MARS5, CEM Corporation, Matthews, NC, USA) set at 200 °C for a 15 min digestion. After the digestion was complete, 10 mL of high-purity water was added after the digestion components had cooled, and the sample was allowed to stand for 30 min after which it was then diluted to a volume of 50 mL. After centrifugation, the supernatant was removed and used in an inductively coupled plasma spectrometer (ICP-OES, Optima8000) to analyze five heavy metal elements (Cu, Zn, Cr, Cd, and Pb). The powder samples of feed, manure, and dust were digested by microwaving for 40 min after adding HNO_3_ (7 mL) and 3 mL hydrochloride (HCl) to the mixture, and the other steps were consistent with those for the PM_2.5_ treatment.

### 2.5. Ultrastructure Observation of the Sample

The PM_2.5_ filter membrane was cut to a size of 1 cm^2^ and directly pasted on a metal post with conductive adhesive, after which the morphology was observed with a scanning electron microscope ([SEM] Hitachi, Tokyo, Japan) after plating with about 20 nm gold. An energy spectrometer (EDS, Bruker, Germany) was connected to the SEM for fixed-point scanning analysis of the individual particles in the sample. The four types of samples were analyzed for the contents of 14 chemical elements (Na, Mg, Al, Si, P, S, Cl, K, Ca, Fe, Ni, Cu, Zn, and Cr). 

### 2.6. DNA Extraction and Bacterial/Fungal Sequencing

After using a gene extraction kit to extract the total DNA of the sample (Biomarker Soil Genomic DNA Kit, RK02005), the V3–V4 region of the bacterial 16SrRNA gene was amplified using primers 338F (5′-ACTCCTACGGGAGGCAGCA-3′) and 806R (5′-GGACTACHVGGGTWTCTAAT-3′) and the fungal ITS1 region primers ITS1F (5′-CTTGGTCATTTAGAGGAAGTAA-3′) and ITS2 (5′-GCTGCGTTCTTCATCGATGC-3′) with added sequencing adapters at the ends for polymerase chain reaction (PCR) amplification. The bacterial amplification program was 95 °C for 5 min followed by 30 cycles of 95 °C for 30 s, 50 °C for 30 s, 72 °C for 60 s, and a final extension at 72 °C for 5 min. The fungal amplification program was 95 °C for 5 min followed by 35 cycles of 95 °C, 50 °C, and 72 °C (all for 1 min), and a final extension at 72 °C for 7 min. The amplified products were purified, quantified, and homogenized to form a sequencing library. The constructed library was first subjected to library quality control, and the qualified library was sequenced on an Illumina HiSeq 2500 platform. The original image data file was converted into original sequenced reads after base calling analysis, and the results were stored in FASTQ file format containing the sequence information (Reads) and the corresponding sequencing quality information. The sequence processing comprised several steps, including quality filtering using the Trimmomatic v0.33 software to filter the raw reads obtained by sequencing followed by the use of Cutadapt 1.9.1 software to identify and remove primer sequences to obtain clean reads without primer sequences. The paired-end sequences were then spliced using Usearch v10 software to splice the clean reads of each sample through overlap, and the spliced data were filtered according to the length ranges of different regions. Finally, chimeric sequences were removed using UCHIME v4.2 software to obtain the final effective data (effective reads).

### 2.7. Data Analysis

SPSS 17.0 (IBM, Armonk, NY, USA) was used for one-way analysis of variance (ANOVA), and Duncan’s test was used for multiple comparison analysis. The results were expressed as mean ± standard deviation. *p* values less than 0.05 (*p* < 0.05) or 0.01 (*p* < 0.01) were taken to indicate statistically significant differences. The heavy metal composition data of PM_2.5_, feed, manure, and dust were processed to represent four source substances in Principal Component Analysis (PCA). This was achieved by Origin 2021b software based on the choice of the components on a scree plot. In addition, the average values of PM_2.5_, NH_3_, H_2_S, and CO_2_ at the same time and in the same place were calculated. These variables were used to perform a Pearson’s correlation analysis and to draw a correlation heat map by Origin 2021b. Furthermore, QIIME (Quantitative Insights Into Microbial Ecology) software was used to select the microbial characteristic sequences with the highest abundance at the genus level as representatives. Multiple sequence alignments were carried out, and phylogenetic trees were constructed. The graphs were drawn using Python (Wilmington, DE, USA) language tools.

## 3. Results and Discussion

### 3.1. Microclimate Variables in the Pig House

The piggery environment monitoring was conducted from 16 March 2021 to 23 March 2021. During the experiment, room temperature and humidity were maintained in a relatively stable state, while the changes in the concentrations of PM_2.5_, NH_3_, CO_2_, and H_2_S in the pig house showed more variation (Table 1). The concentration of PM_2.5_ increased significantly (*p* < 0.05) after feeding in the morning and remained at a high level afterwards. However, no significant change in PM_2.5_ concentration within 1 h after feeding in the afternoon was noted. At the same time, the concentration of NH_3_ was significantly higher than at other periods before feeding in the morning, while the concentration of CO_2_ was the highest at night. The concentration of H_2_S was consistently low, and no significant differences among different time periods were found. Concentrations of several air pollution sources were more stable before feeding in the morning and at night.

At present, there is no reference concerning control standards for the PM_2.5_ concentration in pig houses. However, the concentration of PM_2.5_ in this experiment was lower than in other studies. For example, Shang et al. [13] monitored three fattening pig houses in Beijing for one year and reported concentrations of PM_2.5_ averaging 60–200 μg·m^−3^. This variation may have been due to the low feeding density and timely manure removal. Removing manure frequently is an effective method for reducing gas pollution in the house [14]. Moreover, the feeding density of pigs was 0.502 head m^−2^ in this experiment, which was lower than that in related reports and the density on general commercial pig farms [15]. In addition, the change in PM_2.5_ concentration during daytime was significantly higher than that at night, and significant before and after feeding in the morning. Combined with significant activity observed before and after feeding during the day, this indicated that PM_2.5_ concentration in the pig house was likely to be affected by the activities of farm workers and pigs. Shen et al. [3] found a significant correlation between pig activity and PM concentration in a pig house. When pigs are fed or startled, their activity and PM concentration increase significantly, whereas when pigs are relatively quiet or at rest, the indoor PM concentration is relatively low. In the meantime, the activities of animals and farmers will cause an increase in the airflow in the house, in turn hindering the deposition of particles [16]. Therefore, to reduce the concentration of PM on pig farms, it is necessary to provide pigs with a comfortable and relatively quiet environment to reduce their stress levels.

Among several air pollution sources, CO_2_ is produced during animal respiration, whereas NH_3_ and H_2_S are produced when undigested proteins and S-containing proteins in pig manure decay and deteriorate [17]. The parameters of temperature, humidity, NH_3_ and H_2_S in this experiment (Table 1) met the respective standards of 27 °C, 85%, 25 mg·m^−3^ and 10 mg·m^−3^ given in NY/T 17824.3-2008 “Environmental parameters and environmental management for intensive pig farms”. However, the CO_2_ concentration did not meet the respective standards of 1500 mg·m^−3^ as prescribed by The Ministry of Agriculture of the People’s Republic of China, 2008. In addition, the threshold values of NH_3_, H_2_S, and CO_2_ given by the extension center of Iowa State University in the United States are 8 mg·m^−3^, 8 mg·m^−3^ and 5893 mg·m^−3^, respectively. These are higher requirements for NH_3_ and H_2_S, which have a pungent smell, while being a lower requirement for CO_2_.

The relatively high CO_2_ concentration may have been caused by low ventilation in the experimental cubicle, a situation that is more common in low-ventilation pig houses in autumn and winter [18]. In addition, there was no significant difference in temperature, humidity, or H_2_S concentration within one day. Temperature was artificially controlled to reduce the influence of diurnal temperature range on PM_2.5_ concentration, microbial components, and secondary particle formation in the piggery. Humidity may have an impact on the condensation, deposition, and microbial components of PM_2.5_ [19]. The humidity was relatively stable along with the temperature. H_2_S is a toxic and harmful gas produced by anaerobic bacterial decomposition of sulfur-containing organic matter such as protein. The main source of H_2_S production in the piggery partially overlapped with PM_2.5_ (manure). In addition, the fluctuation in NH_3_ concentration was consistent with the monitoring results of Pu et al. [20]. It is worth noting that the high concentration of NH_3_ in the farm may facilitate reactions with acidic gases (such as H_2_SO_4_, HNO_3_, and HCl) to form secondary particles, such as (NH_4_)_2_SO_4_, NH_4_NO_3_, and NH_4_Cl, while H_2_S may also be a precursor of secondary particles [21]. The reaction intensity of secondary particle transformation is related to the animal species and feeding patterns (such as manure, bedding, and feed type). A relatively high amount of secondary particle formation was found in a chicken house [22], and the formation of secondary particles in pig sheds remains to be determined.

### 3.2. Microscopic Morphology

**Figure 2:**
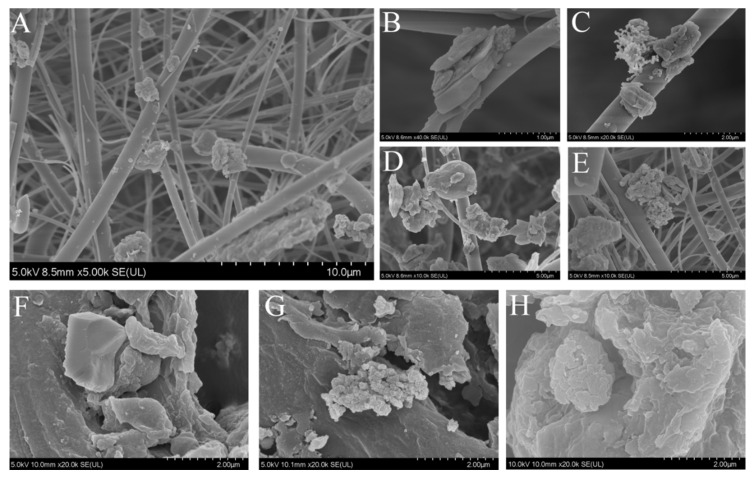
Microscopic morphology of particulate matter with an aerodynamic diameter of less than 2.5 μm (PM_2.5_), feed, manure, and dust. (**A**–**E**): The morphology of PM_2.5_ particles. (**F**,**G**,**H**): electron micrographs of feed, manure, and dust particles.

Many particles either wrapped or absorbed from different pollution sources can fuse together and turn into PM_2.5_ particles of different shapes (Figure 2A–E). In this experiment, the morphology of individual PM_2.5_ particles was divided into four types. The most common were broken, rough, and angular particles (Figure 2E). These particles have rough surfaces, no edges, and many other components that are adsorbed. Spherical particles with smooth surfaces and adsorbing deposited particles were also seen (Figure 2D). In addition, layered fragment-like particles were found (Figure 2B). These particles were angular and smooth. Finally, highly transparent and highly agglomerated grape-like particles were also observed (Figure 2C). These were composed of several circular particles with distinct gaps. At the same time, some environmental sample particles (feed, dust, manure) were prepared by artificial drying and crushing. The angular independent particles (Figure 2F) are often seen in the feed sources and are similar in their morphological characteristics to those shown in Figure 2B. Manure particles are mostly coarse and granular (Figure 2G), and these particles are more common in pig house PM_2.5_ (Figure 2A,E). In addition, the dust particles’ internal gaps were tighter (Figure 2H), and the surface was smoother than that of manure particles. Finally, grape-shaped particles (Figure 2C) with large internal voids were not found in feed, manure, or dust particles. These particles may be formed by other pollution sources.

The same pollution source in a pig house can produce different forms of PM_2.5_ particles. Cambra-López et al. [23] crushed and identified the particles in pig house feed and manure and found that the feed particles were in the form of geometric cubes or were strip or angular broken particles, whereas manure particles were mainly round, smooth, and spherical or broken, rough, and/or angular. This description was similar to the observations in this experiment. Particles of the same substance with different morphologies may be due to the wear and tear of PM_2.5_ during transfer and re-suspension, resulting in shape changes [24]. Moreover, the morphology of all kinds of particles may also change due to the water content. When the moisture content in the particles is higher, the smaller particles will gather and bind to the larger particles [25]. Feed, manure, and dust transformation into PM_2.5_ will be affected by the humidity in the house. The manure is affected not only by the changes of humidity in the house but also by the digestion and metabolism of pigs under the manure management model (for example, whether manure is soaked in water).

### 3.3. Chemical Components

**Figure 3:**
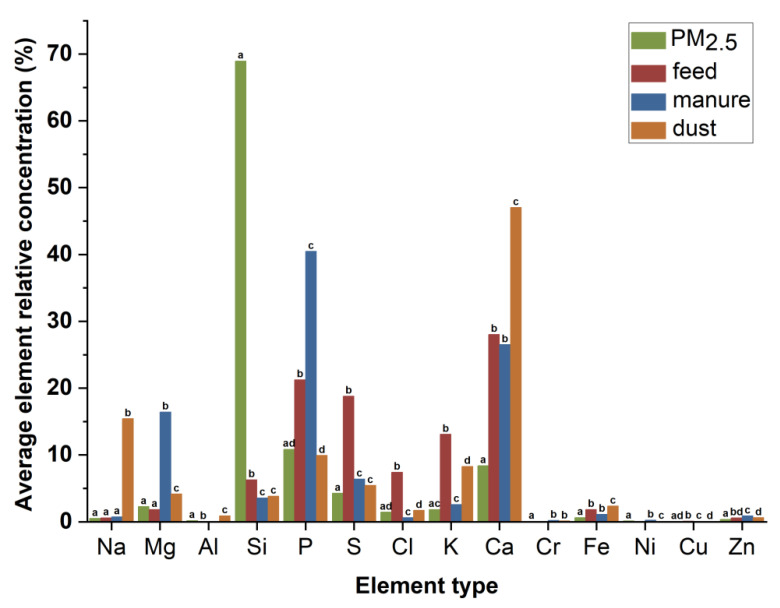
Average element relative concentration (%) for particles from different sources. *n* = 6 for PM_2.5_ and *n* = 3 for others. Averages within an element lacking common superscript letters are significantly different (*p* < 0.05).

Figure 3 shows the mass percentages of 14 elements (Na, Mg, Al, Si, P, S, Cl, K, Ca, Fe, Ni, Cu, Zn, and Cr) found in the PM_2.5_, feed, manure, and dust particles as determined by SEM-EDS. It can be seen from the figure that Na, Mg, Si, P, S, Cl, K, Ca, Fe, and Zn all exist in the four kinds of particles, but the element content concentrations are different between particle types. Content of Na in the dust was the most prominent (15.44%). The contents of Ca and P in the feed (28.05% and 21.26%, respectively) and manure (26.52% and 40.47%, respectively) were higher, but the feed also contained higher S (18.81%) and K (13.10%). Furthermore, the Al in the feed was only 0.03%, but Cr did not reach the lower detection limit, and Al was not detected in the manure while Cr was detected in the manure (0.23%). In addition, the Si concentration of PM_2.5_ was very high, which may be due to the influence of a large number of Si elements in the quartz filter membrane used in this experiment and thus Si was not included in the follow-up analysis.

The specific values of five trace metals (Cu, Zn, Cr, Cd, and Pb) in PM_2.5_, feed, manure, and dust were further determined by inductively coupled plasma optical emission spectrometry (ICP-OES) as shown in Table 2. Although the Zn content was the highest in the four samples, large differences in the content of Cr and Cu among different samples were found. The contents of Cd and Pb in all samples were low. The Cd of feed was 0.02–0.08 mg·kg^−^^1^, and the Pb was not up to the detection limit.

Heavy metals in particulates can accumulate in the respiratory system and can be harmful to health. Moreover, some trace metals (Cu, Zn, Cr, Cd, or Pb) can indicate the source of particle pollution due to their stability and relatively simple sources in the natural environment [26]. The contents of Zn and Cu in the feed were high, and both were also high in PM_2.5_. This may play a supporting role in the previous argument that PM_2.5_ in pig houses mainly arises from the feed and feed metabolites (manure) [27]. However, only a low content of Cr was detected in the feed and manure, but the content of Cr in PM_2.5_ was higher than that of Cu, while the contents of Cu in feed, manure, and dust were significantly higher than that of Cr. This may be due to the accumulation in the process of material transformation, or it may be due to the influence of other pollution sources. Wang et al. [28] had encountered a similar situation, and they speculated that the material outside the house might be one of the sources contributing to PM_2.5_ inside the house. In addition, PM_2.5_ contained elements such as Na, Mg, Al, P, S, CI, K, Ca, and Fe. Mg, P, and K were detected in manure; Ca was mainly from feed; Na and Cl were detected in particles from sawdust; and S was detected in particles from skin and feed [29,30,31]. The contents of Na in the dust were observed significantly higher than those in the feed, and manure, and PM_2.5_, this may be due to the dust in the piggery, including outside resources carried by the breeding workers. Thus, combined with the results of previous studies, the sources of Mg, K, Ca, P, S in the PM_2.5_ were from feed and manure.

### 3.4. Water-Soluble Ions of PM_2.5_

**Figure 4:**
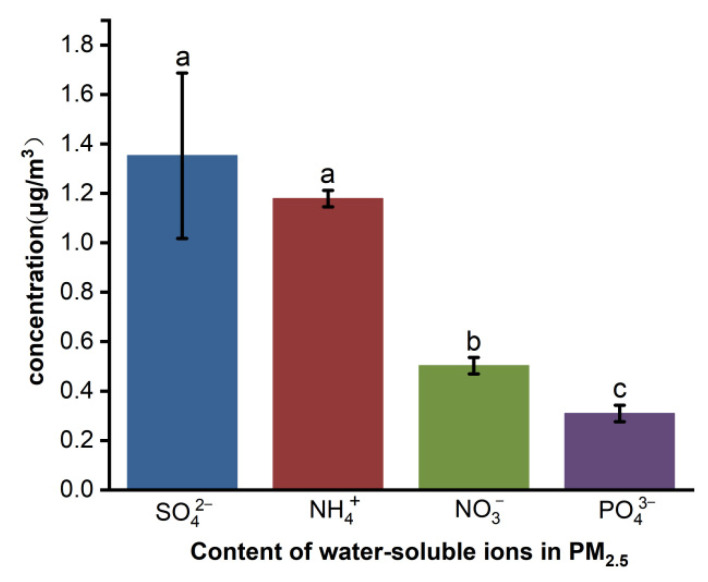
The contents of several water-soluble ions in PM_2.5_. Note: Different superscript letters in the same column represent significant variation (*p* ≤ 0.05), while the same letters represent no significant variation (*p* > 0.05).

The contents of several water-soluble ions in PM_2.5_ are shown in Figure 4. The average concentrations of NH_4_^+^, SO_4_^2−^, and NO_3_^−^ were 1.18, 1.26, and 0.50 μg∙m^−3^, respectively. In this experiment, the concentration of NO_3_^−^ was significantly lower than NH_4_^+^ and SO_4_^2−^, but higher than PO_4_^3−^ (0.31 μg∙m^−3^). If calculated according to the average concentration of PM_2.5_ during the experimental period (41.63 μg∙m^−3^), NH_4_^+^, SO_4_^2−^, NO_3_^−^, and PO_4_^3−^ accounted for 2.83%, 3.03%, 1.27%, and 0.74% of the mass of PM_2.5_, respectively.

Ions of NH_4_^+^, SO_4_^2−^, and NO_3_^−^ are the main water-soluble ions in pig house PM_2.5._ These three substances belong to secondary ions that are not only directly discharged but are also formed in the environment. They are important substances affecting the formation of particles [32]. The high concentration of NH_3_ in pig houses is conducive to the formation of NH_4_^+^, which leads to a higher concentration of NH_4_^+^ in PM_2.5_, and the secondary source of SO_4_^2−^ is the photochemical reaction and liquid phase oxidation of SO_2_. Khoder et al. [33] suggested that the higher SO_4_^2−^ and NO_3_^−^ contents in PM_2.5_ in summer were related to the high rate of photochemical activity and high temperature. In general, the SO_2_ concentration in pig houses is lower than that of NH_3_, but the concentration of SO_4_^2−^ in PM_2.5_ is not significantly different from that of NH_4_^+^. It is likely that there were some substances in the pig house that reacted easily with SO_2_, making it easier than NH_3_ to transform into secondary particles. In addition, a large part of NO_3_^−^ in PM_2.5_ comes from the reaction of HNO_3_ gas produced by NOx conversion with NH_3_ in air to form NH_4_NO_3,_ and part of the NO_3_^−^ will also exist in the form of Ca(NO_3_)_2_ or Mg(NO_3_)_2_ [34].

### 3.5. Microbial Components of PM_2.5_

**Figure 5:**
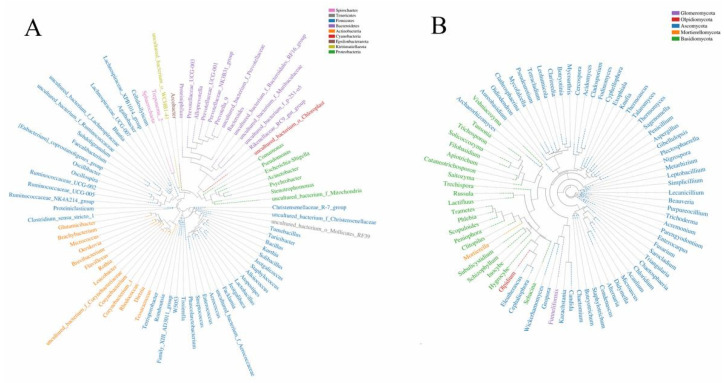
The evolutionary tree of bacterial and fungal species in PM_2.5_. (**A**) The evolutionary tree of bacterial species. (**B**) The evolutionary tree of fungal species. Note: The same color represents the same phylum. Each branch in the evolutionary tree represents a species, and the length of the branch represents the evolutionary distance between the two species, for example, the degree of species difference.

According to the Good’s coverage (>96%), the sequences produced by Illumina sequencing captured most of the bacteria and fungi in the PM_2.5_ samples. Alpha diversity measures showed that the average Shannon index and Chao 1 index of bacteria were 6.93 and 947.27, and the corresponding indexes of fungi were 7.01 and 775.07, respectively. This indicated that the diversity (Shannon index) of bacteria and fungi in PM_2.5_ was similar, while the abundance (Chao 1 index) of bacteria was higher than that of fungi. A total of 24 phyla and 378 genera of bacteria were classified from three PM_2.5_ samples. Firmicutes was the most dominant phylum, followed by Actinobacteria, Bacteroidetes, and Proteobacteria. These four phyla accounted for 95.92–96.68% of the total sequences, indicating that bacteria mostly belonged to these four phyla at the genus level (Figure 5A). Furthermore, 12 phyla and 380 genera were extracted from fungi. Ascomycota and Basidiomycota phyla accounted for 72.54–87.05% of the total sequences. Most of the fungi with high abundance at the genus level belonged to these two phyla (Figure 5B). Therefore, combining A and B in Figure 5, we can see that although fungi had similar diversity to bacteria at the genus level, the dominant fungi almost all belonged to Ascomycota and Basidiomycota, and the diversity at the phylum level was not as high as in bacteria.

At the genus level, 15 dominant genera were detected (relative abundance of at least one sample > 1%). *Clostridium_sensu_stricto_1* and *Lactobacillus* were the most abundant (abundance of 9.25–12.29%) followed by *Corynebacterium_1* and *Turicibacter* (abundance of 3.86–7.12%). Interestingly, several bacteria with the highest abundance in this experiment (*Clostridium_sensu_stricto_1*, *Lactobacillus*, *Turicibacter* etc.) have been demonstrated to be related to manure microorganisms [35]. *Lactobacillus* was more commonly found in pig intestines and manure [36]. *Clostridium* was enriched in the small intestine of pigs [37]. Among the other dominant bacteria, *Terrisporobacter* and *Romboutsia* were deemed to be crucial genera degrading organic matter [38]. Combined with previous studies, this suggested that manure was the main source of bioaerosols in pig houses.

In addition, many types of pathogens were found in the pig house PM_2.5_. According to the directory of pathogenic microorganisms infecting humans published by the Ministry of Health of the People’s Republic of China (MOHC), a total of five pathogenic bacteria genera were identified (*Acinetobacter*, *Pseudomonas*, *Streptococcus*, *Escherichia-Shigella*, and *Staphylococcus*). In terms of fungi genera, *Mortierella* was the highest (8.99–20.16%) followed by *Scopuloides* (0.53–5.03%). According to 123 fungal allergenic genera listed by Birgit et al. [39], six allergenic genera (*Aspergillus*, *Schizophyllum*, *Wallemia*, *Trichosporon*, *Sporobolomyces*, and *Trichoderma*) had a relative abundance > 0.01%. Most of these pathogens or allergens have been identified in past studies. For instance, White et al. [40] identified 28 different microbial pathogens, including *Filobasidium uniguttulatum*, *Bacillus cereus*, *Aerococcus viridans*, and *Enterococcus avium*. Viegas et al. [41] identified a variety of fungal allergens from *Aspergillus*, *Fusarium*, *Penicillium*, and other genera in an animal house. Although most of the bacteria and fungi identified in this experiment are considered non-pathogenic, the high concentration of PM in pig houses can provide more attachment points for microorganisms in the air and promote their reproduction [42]. This process causes an increase in the inflammatory potential of PM_2.5_. Zhiping et al. [43] demonstrated that an increase in mold and bacteria concentrations in the air are associated with airway inflammation and general deterioration of lung function. Moreover, due to the extremely small particle size, PM_2.5_ can easily carry a variety of microorganisms that are deposited into respiratory bronchioles and alveoli during gas exchange and subsequently penetrate the lungs and travel into the blood circulation [44]. Tang et al. [45] found that the bacterial composition in a pig house’s PM_2.5_ was very similar to those in pig bronchi and lungs. Even if PM_2.5_ is excluded with ventilation, it will stay in the atmosphere for a long time and can be dispersed over longer distances [46]. Dee et al. [47] detected porcine reproductive and respiratory syndrome virus RNA and *Mycoplasma hyopneumoniae* DNA in air samples at a distance of 4.7 km from the pig house, thus posing a serious health threat to the residents around the farm. Therefore, it is of concern that the bacterial components of PM_2.5_ from the piggery can enter the respiratory system and may pose potential health risks to humans and animals.

### 3.6. Correlation with Variables

#### 3.6.1. The Correlation between PM_2.5_ and NH_3_

**Figure 6:**
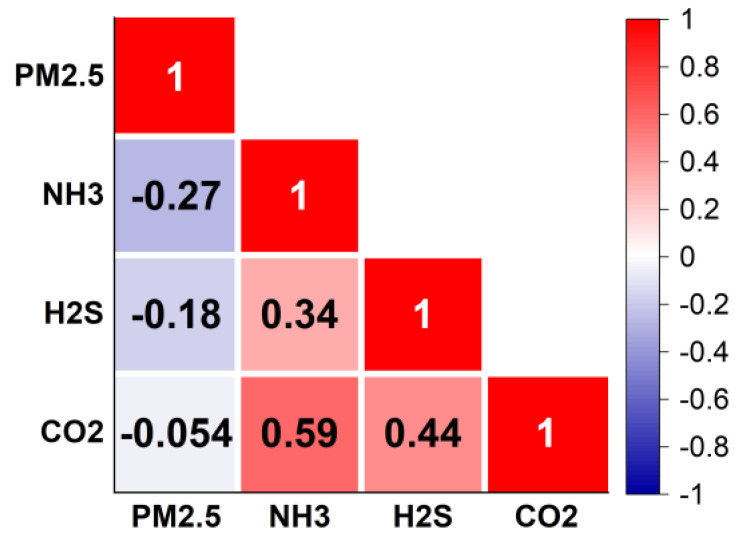
Correlations between PM_2.5_ concentration and some environmental parameters.

A correlation analysis for PM_2.5_, NH_3_, CO_2_, and H_2_S concentrations is presented in Figure 6. The change in PM_2.5_ concentration was not strongly correlated with NH_3_, CO_2_, or H_2_S, which are the common environmental indicators in pig houses. PM_2.5_ had negative correlations of 0.27 and 0.18 with NH_3_ and H_2_S, respectively. Among all indicators, the correlation between concentrations of NH_3_ and CO_2_ was the strong (+0.59) followed by correlations between NH_3_ and H_2_S (+0.44), respectively.

The significant correlation between the concentration of NH_3_ and CO_2_ was similar to those reported in previous studies [48,49]. This correlation may stem from a series of chemical reactions. When water (H_2_O) exists in the environment, CO_2_, NH_3_, and H_2_O can react to form (NH_4_)_2_CO_3_ or NH_4_HCO_3._ These carbonates are unstable and easily decompose spontaneously. In this experiment, the humidity in the air of the piggery was relatively high, so the reaction among NH_3_, CO_2_ and H_2_O could have occurred, resulting in the change trends of NH_3_ and CO_2_ being similar.

NH_3_ is the precursor of secondary PM generation, and this compound was not highly correlated with PM_2.5_ concentration. Dai et al. [2] observed similar results in a fattening pig house in southern China, a result that may have been due to the low conversion intensity of NH_3_ in the barn environment and the high concentration of NH_3_ as a precursor of alkaline reactions in the barn. However, other substances combine with NH_3_ (such as nitric dioxide [NO_2_] and sulfur dioxide [SO_2_]), and in the pig house environment, present in amounts far lower than those required for the reaction of NH_3_, resulting in the above-mentioned phenomenon. In the results for water-soluble ions (Figure 4), NH_4_^+^ and SO_4_^2−^ only accounted for 2.83% and 3.03% of the PM_2.5_ mass concentration, respectively, but their contents were significantly higher than those of NO_3_^−^ and PO_4_^3−^ (1.27% and 0.74%, respectively), further indicating that secondary particle transformation existed in the pig house, but the transformation intensity was very low. Hristov et al. [50] found that secondary particles emitted by livestock and poultry farming accounted for 5% to 11% of the total PM_2.5_ emissions in the United States, but the contribution of PM_2.5_ directly emitted by livestock farming to the total emissions was < 0.1%. Therefore, compared with in-house transformation, NH_3_ generated in the pig house is more likely to enter the atmosphere with ventilation discharge and produce secondary particles such as NH_4_NO_3_ in the atmospheric troposphere, thus affecting the farm and surrounding areas [51].

#### 3.6.2. The Correlations between PM_2.5_ and Environmental Samples (Feed, Manure, and Dust)

**Figure 7:**
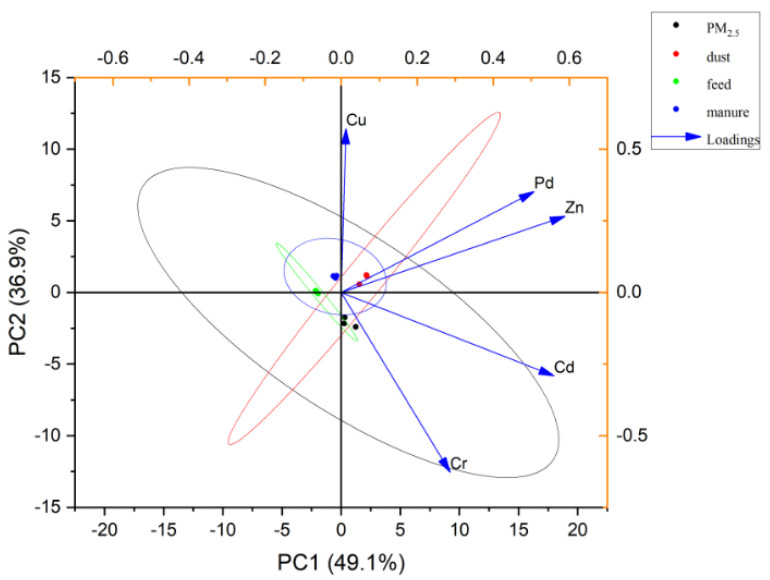
Principal component analysis based on trace metal contents of PM_2.5_, feed, manure, and dust.

Fixed values of trace metals in PM_2.5_, feed, manure and dust were used for PCA, after Cu, Zn, Cr, Cd were selected by a scree plot. Figure 7 shows the results of the analysis, with black, red, green, and blue representing PM_2.5_, dust, feed, and manure, respectively. The distance between two different points indicates their differences, and the ellipse lines of different colors are within the 95% confidence interval (CI) of each other. There are intersecting and overlapping confidence intervals between feed and manure, and this was consistent with the actual metabolism of pigs. Moreover, both were within the confidence interval of PM_2.5_, indicating that feed and manure were likely to be the sources of PM_2.5_ in the pig house. In addition, the CI range of dust overlapped with those of feed, manure, and PM_2.5_ but was not fully contained, indicating that some dust may be one of the sources of PM_2.5_ in the pig house.

**Figure 8:**
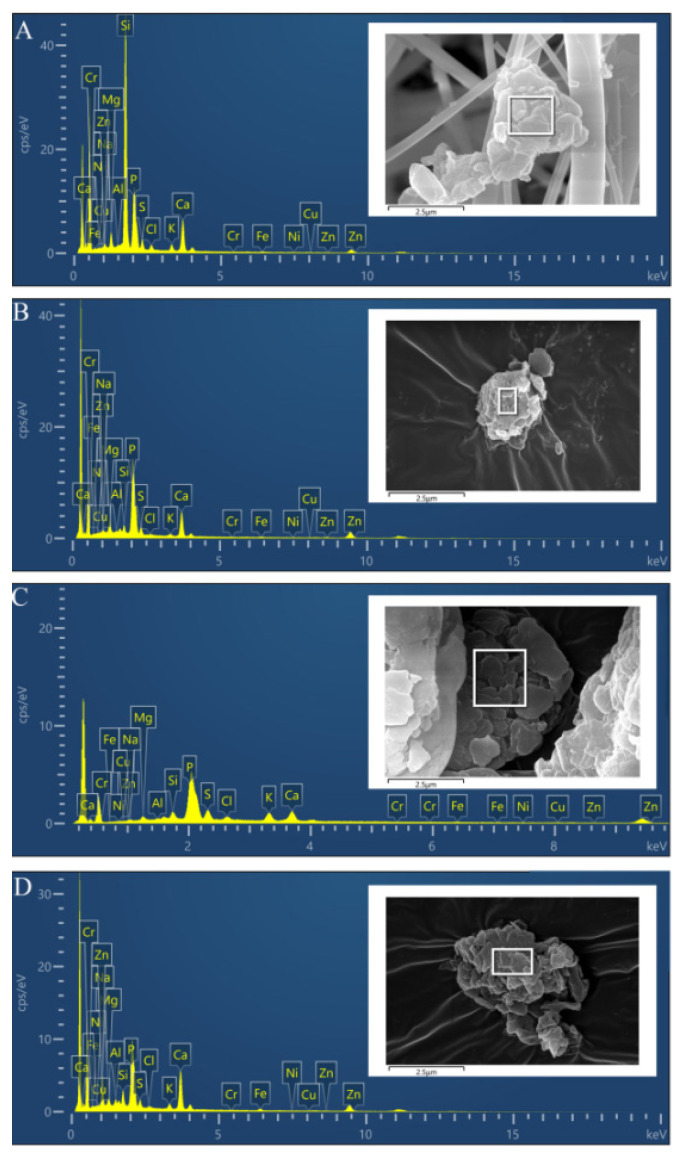
Energy spectrum analysis diagram of oval sedimentary PM_2.5_ and manure particles. (**A**) Oval sedimentary particles formed separately in PM_2.5_ in the pig house. (**B**) Manure pellets. (**C**) Feed pellets. (**D**) Dust pellets.

Combining the morphology and elemental composition of particles from different sources with PM_2.5_ analysis can better reflect the relationship between them. In this experiment, the most common morphologies found in PM_2.5_ in the pig house was rough and angular particles. Figure 8 shows the morphological characteristics and elemental composition of rough and angular particles of PM_2.5_ (Figure 8A) and manure, feed, and dust particles (Figure 8B–D, respectively). In the figure, both manure and dust particles show the morphological characteristics of a rough surface, gaps in the particles, and no sharp edges at the margins. These characteristics are similar to the most common PM_2.5_ morphology. However, the Na content of dust (15.44%) was much higher than that of PM_2.5_ (0.51%), so the proportion of dust to PM_2.5_ in the pig house may be less. After excluding Si (influenced by quartz film), the counts per second per eV of P, Ca, Cl, Zn, and other major elements contained in the two particles shown in Figure 8A and B were similar. Therefore, manure in the pig house is likely to be one of the main contributing sources of PM_2.5_.

Based on the results of principal component analysis and morphological and elemental analyses, it can be inferred that the potential sources of PM_2.5_ in pig houses are mainly feed and manure, with a small amount of dust and other sources. This finding is consistent with the conclusion of Cambra-López et al. [23]. Therefore, removing manure twice a day in pig houses can shorten the retention time of manure, and potentially reducing the PM_2.5_ produced by manure. Moreover, routine use of dry feeders may help reduce PM_2.5_ levels in pig houses compared with pigs that have free access to feed. In addition, as excrement is a metabolite of pigs after eating, its composition is affected by the metabolic rates of the pigs and the composition of feed. Kabelitz et al. [52] showed that the manure particle release potential of finishing pigs was three times that of sows and piglets. This difference may be due to the metabolic intensity and digestive system maturity of pigs at different ages. In addition, the feed ratio is also of concern since sows and piglets have a higher proportion of protein and crude fiber, while finishing pigs have a higher fat content that is more likely to release particles. Cheng et al. [53] confirmed that the use of fresh fermented soybean meal can improve the utilization of dietary nitrogen in piglets, reduce nitrogen excretion, and reduce NH_3_ and PM concentrations in livestock.

## 4. Conclusions

In this experiment, the relationship between PM_2.5_ production in a pig house and feed, manure, dust, and selected air pollutants in the house was studied under an environment designed to reduce the influence of factors outside the house and reduce the temperature difference inside the house. Several conclusions were drawn:

1. Part of the PM_2.5_ in the piggery derives from secondary particles, but this proportion is very small.
2. Most of the PM_2.5_ in the pig house is produced in the form of primary particles, and feed and manure are the main sources.
3. The manure in the pig house can produce elliptical-deposited particles that are common in PM_2.5_ of pig houses and that easily adhere to other components. In addition, manure has an impact on PM_2.5_ concentrations and microbial components. Therefore, the relationship between manure management in pig houses and PM_2.5_ is worth further study in the future.
4. Frequent manure removal and a well-maintained feeding system can help reduce PM_2.5_ concentration in a pig house and the surrounding area.

## Figures and Tables

**Table 1:** Summary data from monitoring of the pig house at different time periods.

Item		PM_2.5_ (μg·m^−3^)	NH_3_ (mg·m^−3^)	H_2_S (mg·m^−3^)	CO_2_ (mg·m^−3^)	Humidity (%)	Temperature (°C)
Before feeding in the morning (7:00–8:00)	Mean ± SD	34 ± 9 ^ac^	19.48 ± 1.71 ^a^	0.28 ± 0.35	2020 ± 209 ^a^	64.48 ± 3.89	24.25 ± 1.28
	Max	52	22.05	0.84	2283	70.94	25.94
	Min	18	16.97	0	1758	59.02	22.52
After feeding in the morning (9:00–10:00)	Mean ± SD	57 ± 26 ^b^	16.16 ± 3.49 ^b^	0.21 ± 0.31	2066 ± 263 ^a^	65.45 ± 4.34	24.59 ± 1.10
	Max	136	18.97	0.78	2463	72.84	26.19
	Min	28	9.32	0	1773	58.21	22.68
Before feeding in the afternoon (14:00–15:00)	Mean ± SD	50 ± 19 ^b^	17.63 ± 4.58 ^b^	0.47 ± 0.46	2341 ± 468 ^a^	64.90 ± 4.93	25.46 ± 1.23
	Max	82	21.54	1.06	2894	72.05	26.89
	Min	19	8.94	0	1512	56.72	23.47
After feeding in the afternoon (16:00–17:00)	Mean ± SD	58 ± 20 ^b^	14.97 ± 4.05 ^b^	0.15 ± 0.20	1870 ± 485.53 ^ab^	66.21 ± 4.71	25.54 ± 1.10
	Max	92	19.79	0.52	2732.18	85.56	26.94
	Min	24	9.43	0	1130.53	56.91	23.79
Rest at night (20:00–21:00)	Mean ± SD	39 ± 16 ^c^	17.17 ± 2.80 ^b^	0.53 ± 0.38	2530 ± 275 ^b^	66.73 ± 4.67	25.06 ± 1.09
	Max	76	21.05	1.14	2912	74.39	26.49
	Min	10	12.88	0	2125	59.38	23.44

Note: Different superscript letters in the same column represent significant variation (*p* ≤ 0.05), while the same letter represents no significant variation (*p* > 0.05). SD, standard deviation.

**Table 2:** Summary of quantitative determination results of heavy metals.

Sample	Cu	Zn	Cr	Cd	Pb
dust (mg·kg^−1^)	82.3 ± 9.4 ^a^	1290.7 ± 71.0 ^b^	13.01 ± 0.14 ^c^	0.35 ± 0.02 ^d^	1.90 ± 0.53 ^d^
feed (mg·kg^−1^)	43.3 ± 5.3 ^a^	139.3 ± 3.1 ^b^	2.75 ± 0.05 ^c^	0.05 ± 0.03 ^d^	ND
manure (mg·kg^−1^)	156.3 ± 9.7 ^a^	610.9 ± 18.7 ^b^	8.96 ± 1.12 ^c^	0.22 ± 0.04 ^d^	0.30 ± 0.14 ^d^
PM_2.5_ (ng·m^−3^)	20.8 ± 4.2 ^a^	548.8 ± 90.0 ^b^	44.84 ± 6.72 ^c^	0.41 ± 0.09 ^d^	0.15 ± 0.06 ^d^

The values are shown as mean ± standard deviation (SD,), *n* = 3. Means within each row followed by different superscript letters were significantly different (*p* < 0.05).

## Data Availability

The data that support the findings of this study are available from the corresponding author, upon reasonable request.
